# Supplementary material for: Fumarate Hydratase Enhances the Therapeutic Effect of PD-1 Antibody in Colorectal Cancer by Regulating PCSK9
Source: Cancers (Basel). 2024 Feb 8;16(4):713. doi: 10.3390/cancers16040713 (PMC10887080; doi:10.3390/cancers16040713)

Figure 1G

FH

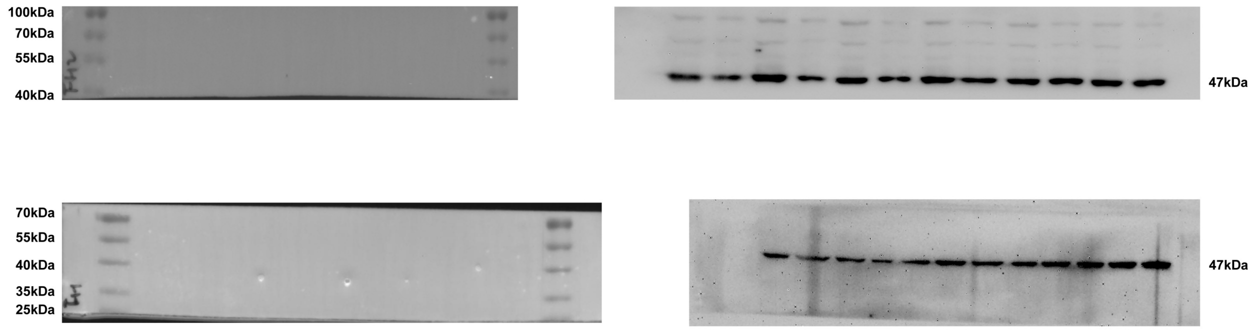

β-actin

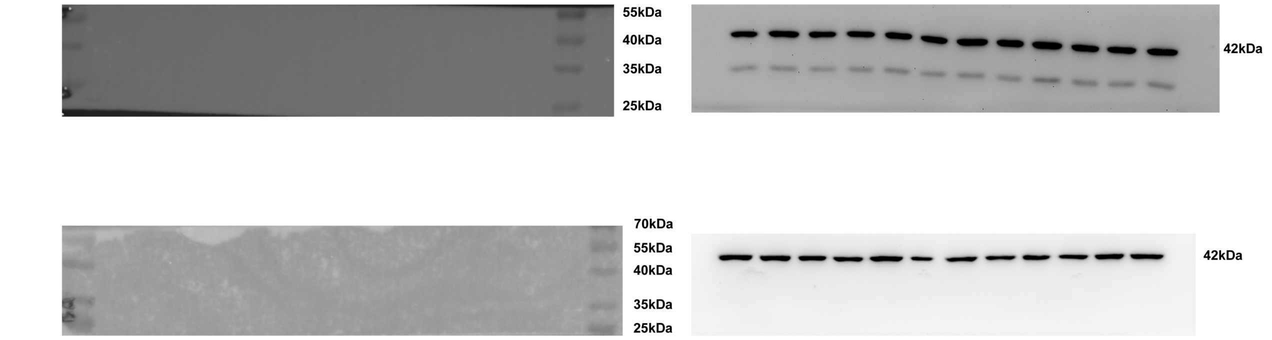

Figure 2A

FH

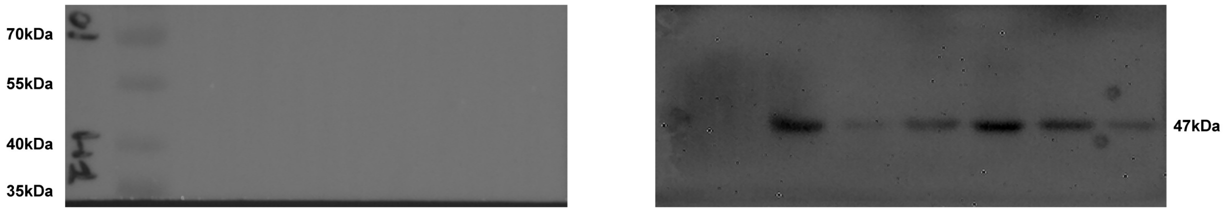

β-actin

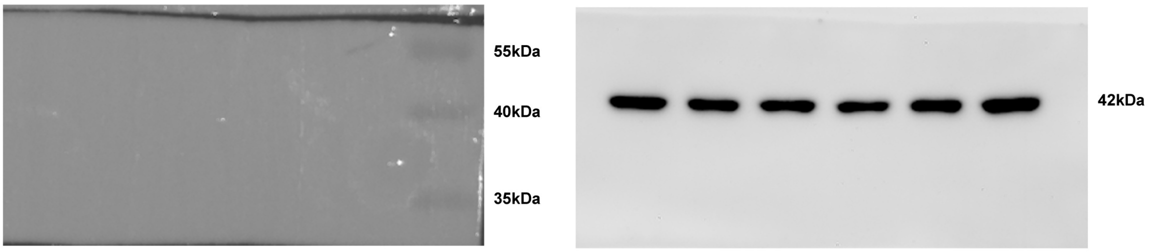

Figure 2B

FH

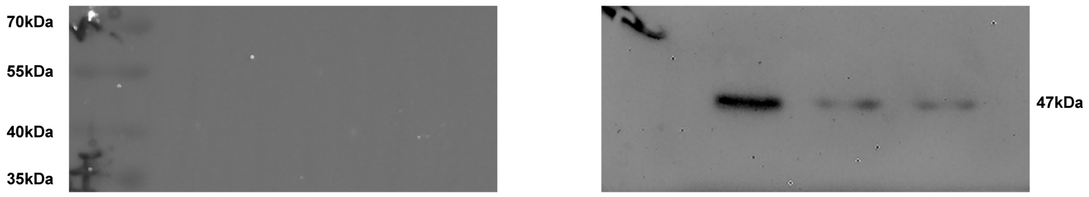

β-actin

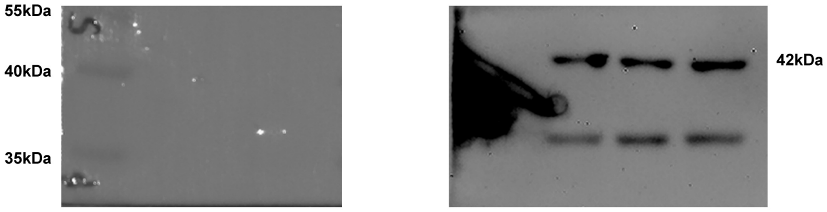

FH

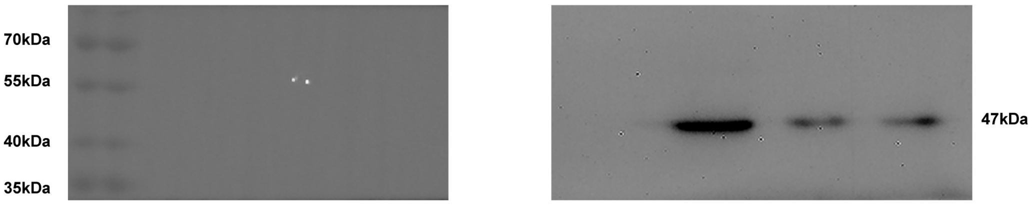

β-actin

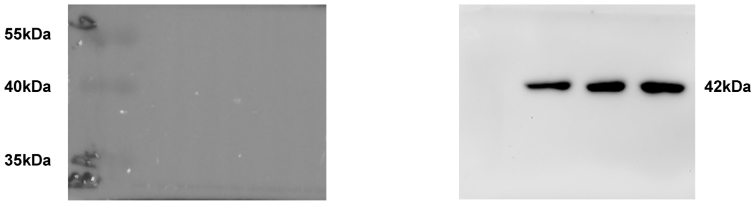

Figure 2J

Myc

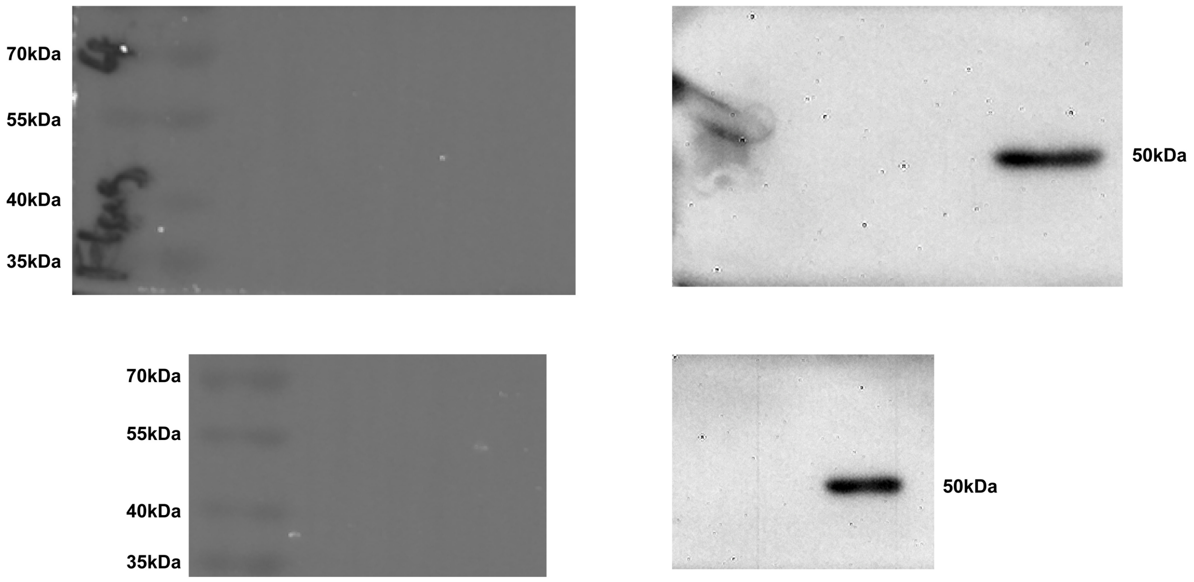

β-actin

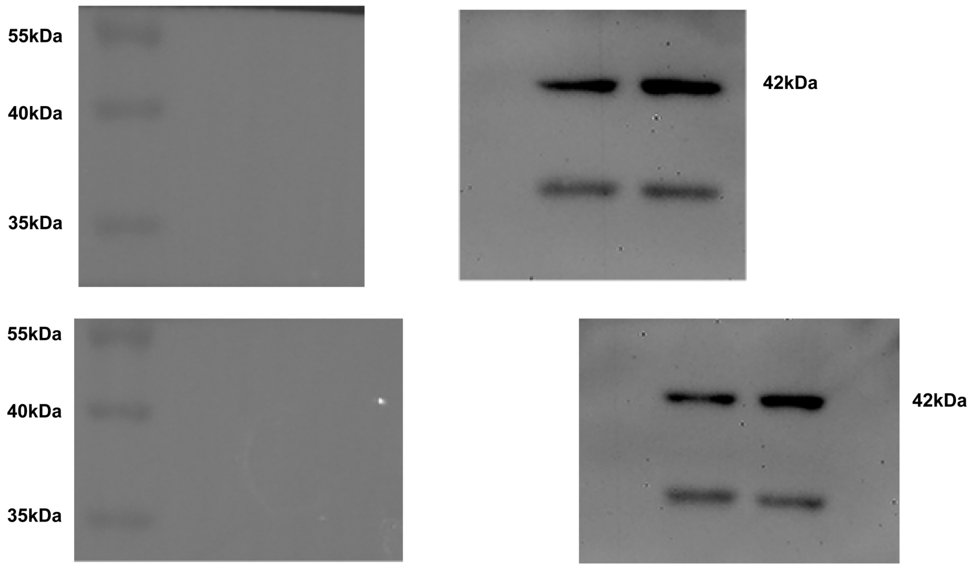

Figure 3A

FH

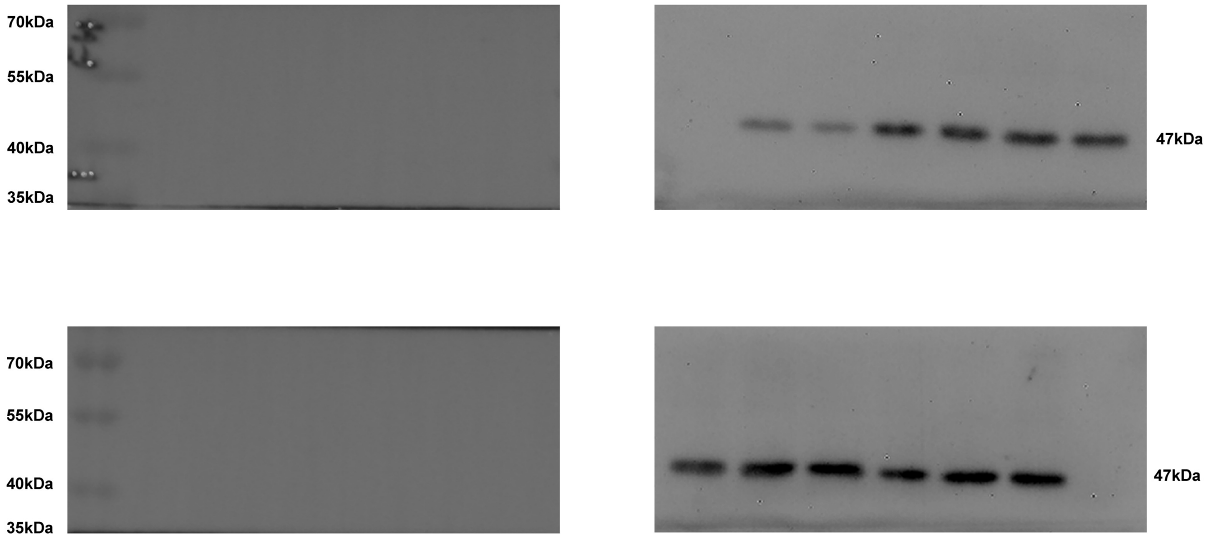

β-actin

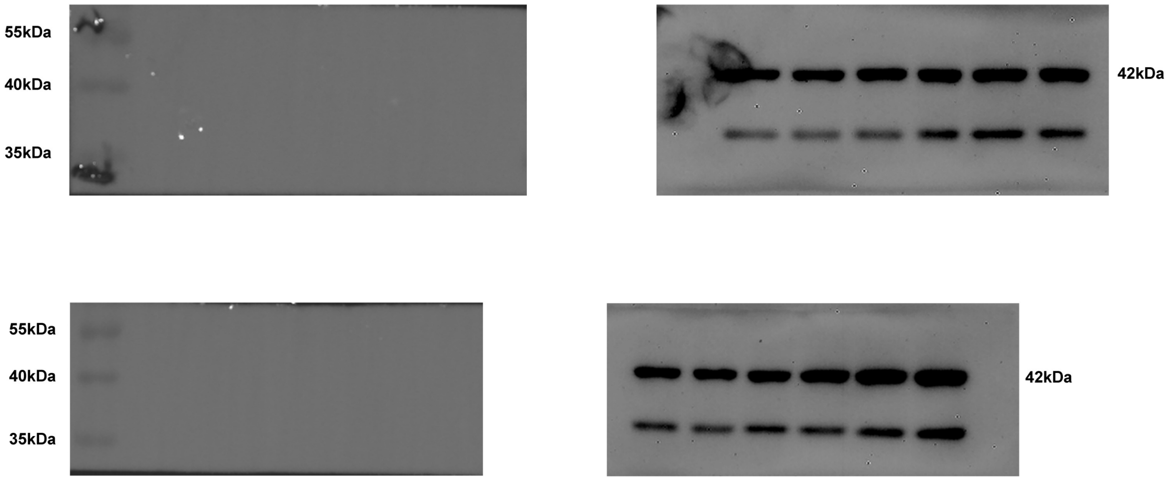

Figure 4B

FH

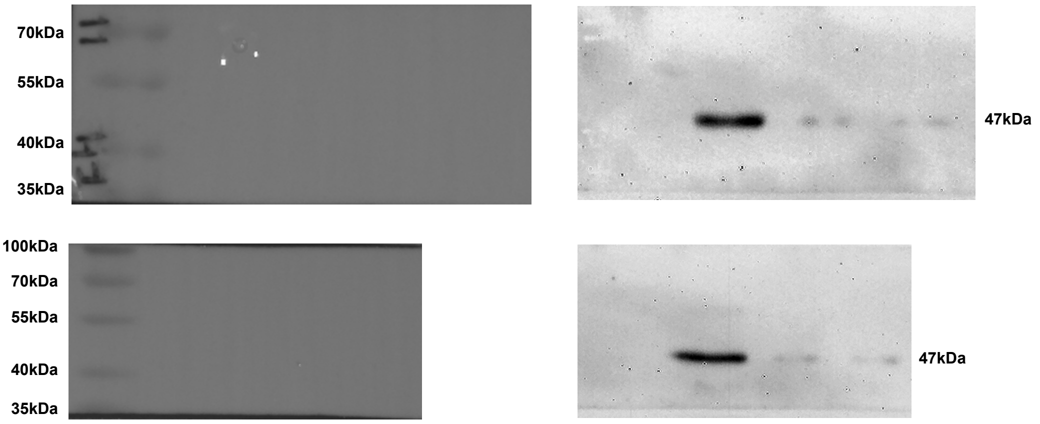

PCSK9

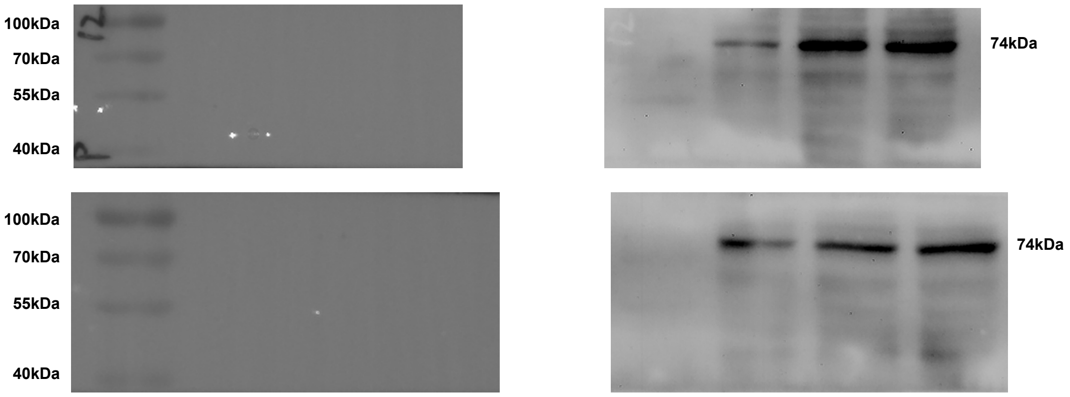

β-actin

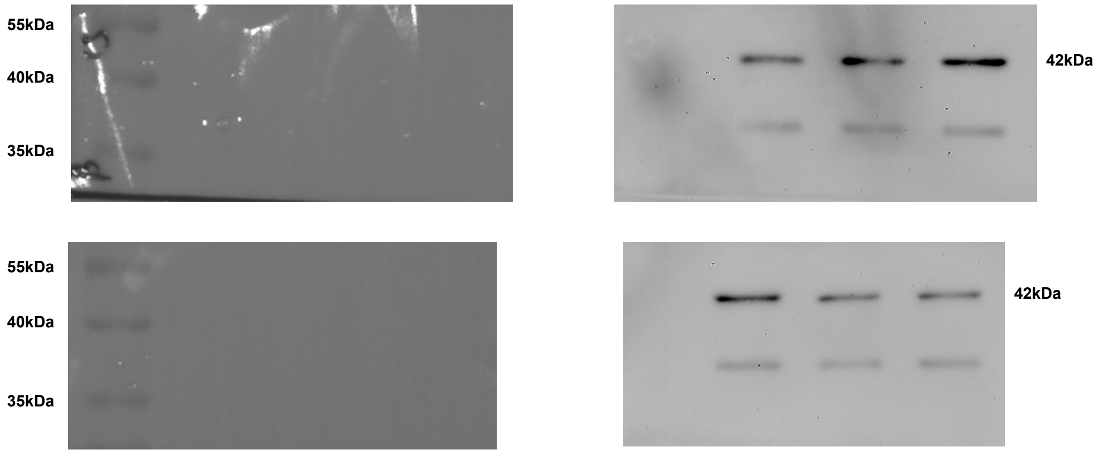

Figure 4D

Myc

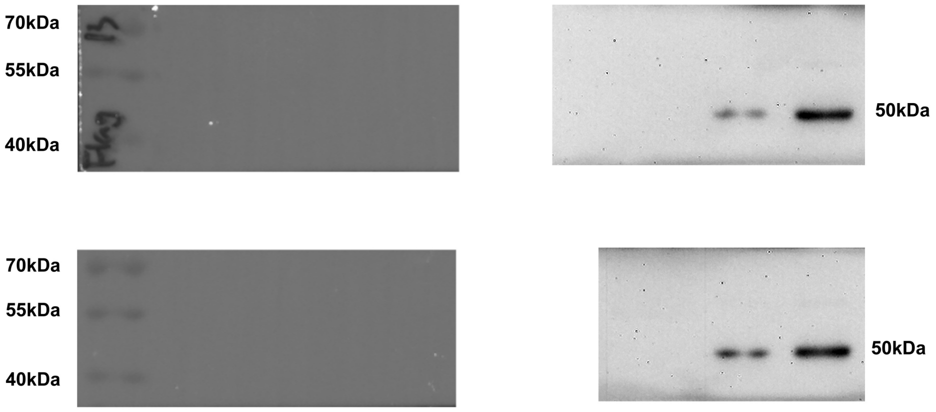

PCSK9

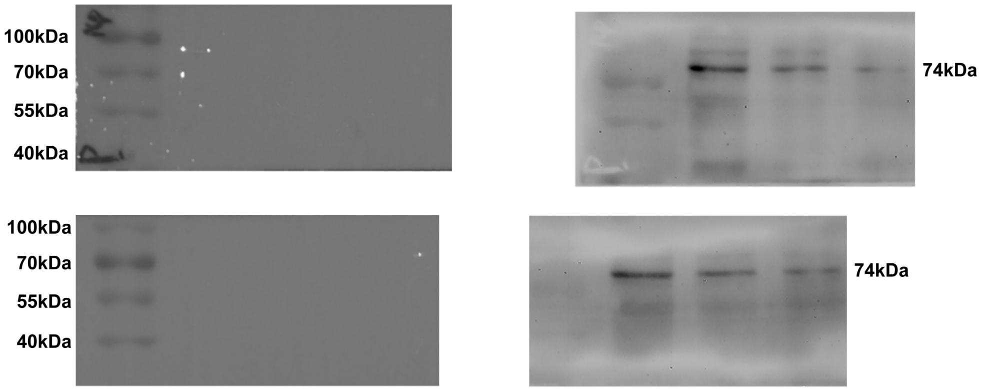

β-actin

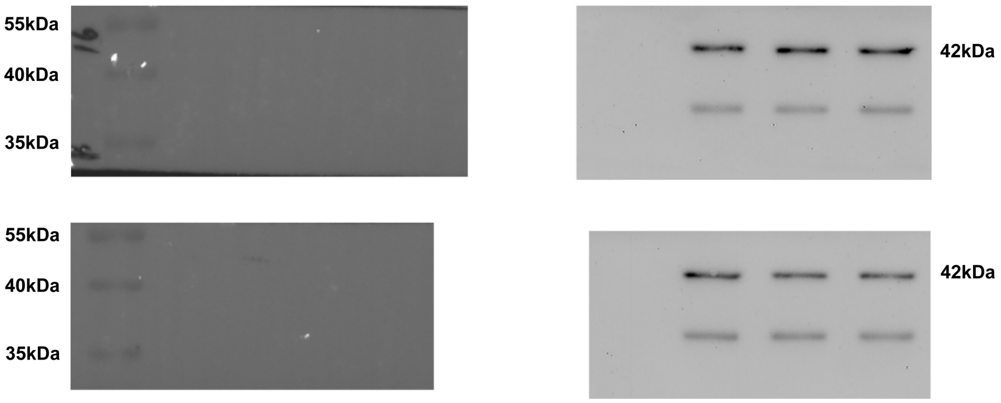

Figure 5E

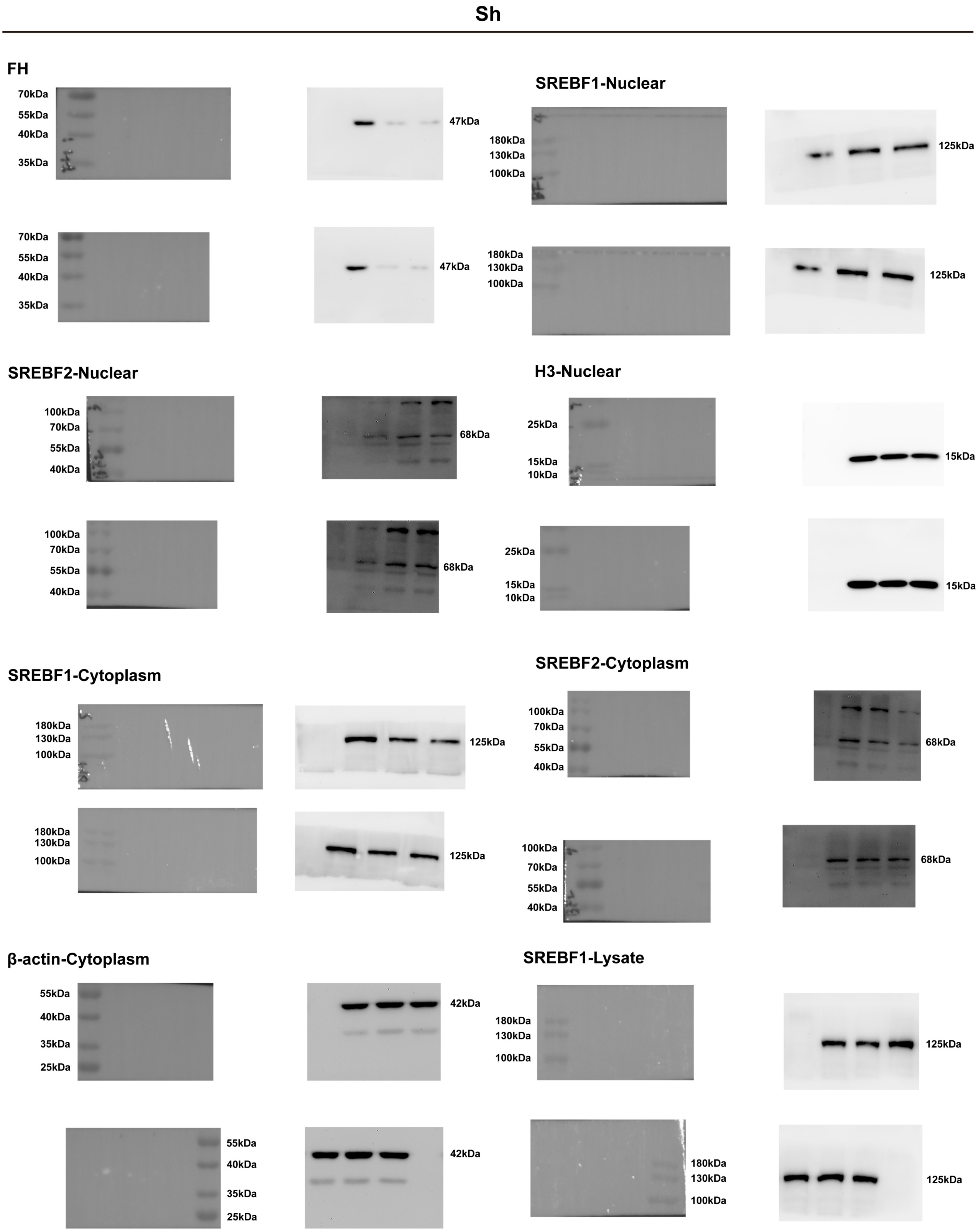
Sh
FH
70kDa
55kDa
40kDa
35kDa
47kDa
70kDa
55kDa
40kDa
35kDa
47kDa
SREBF1-Nuclear
180kDa
130kDa
100kDa
125kDa
180kDa
130kDa
100kDa
125kDa
SREBF2-Nuclear
100kDa
70kDa
55kDa
40kDa
68kDa
100kDa
70kDa
55kDa
40kDa
68kDa
H3-Nuclear
25kDa
15kDa
10kDa
15kDa
25kDa
15kDa
10kDa
15kDa
SREBF1-Cytoplasm
180kDa
130kDa
100kDa
125kDa
180kDa
130kDa
100kDa
125kDa
SREBF2-Cytoplasm
100kDa
70kDa
55kDa
40kDa
68kDa
100kDa
70kDa
55kDa
40kDa
68kDa
β-actin-Cytoplasm
55kDa
40kDa
35kDa
25kDa
42kDa
55kDa
40kDa
35kDa
25kDa
42kDa
SREBF1-Lysate
180kDa
130kDa
100kDa
125kDa
180kDa
130kDa
100kDa
125kDa

SREBF2-Lysate

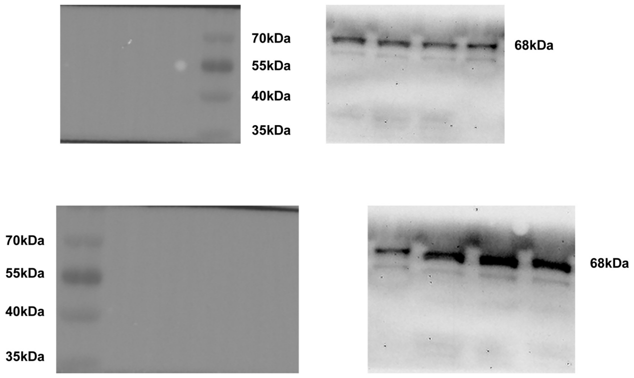

β-actin-Lysate

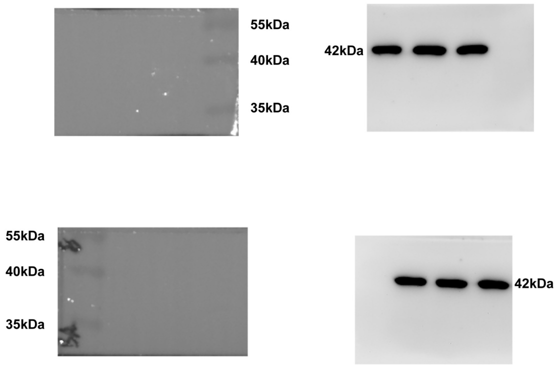

Myc

FH

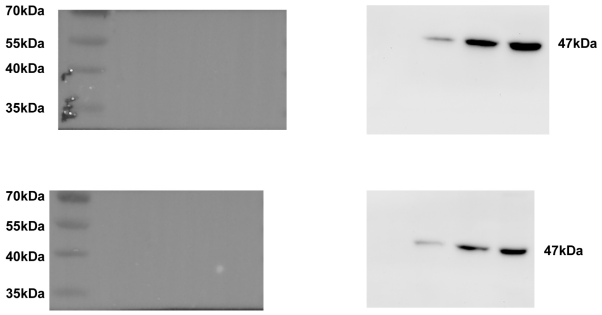

SREBF1-Nuclear

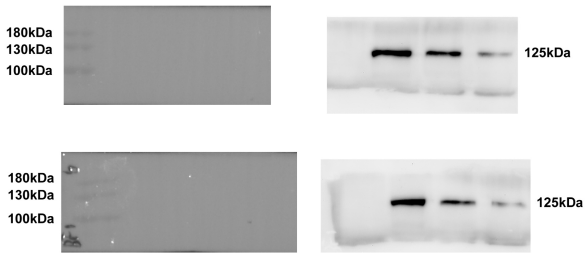

SREBF 2-Nuclear

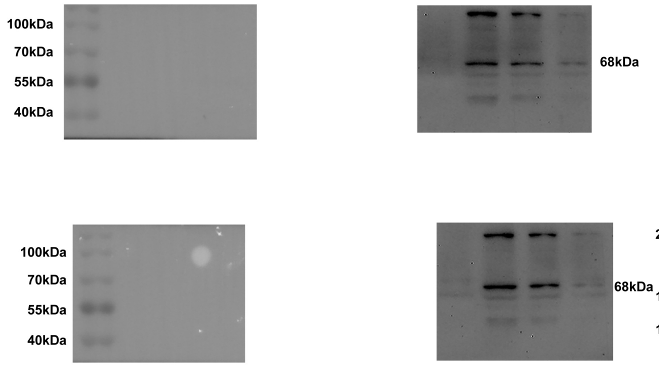

H3-Nuclear

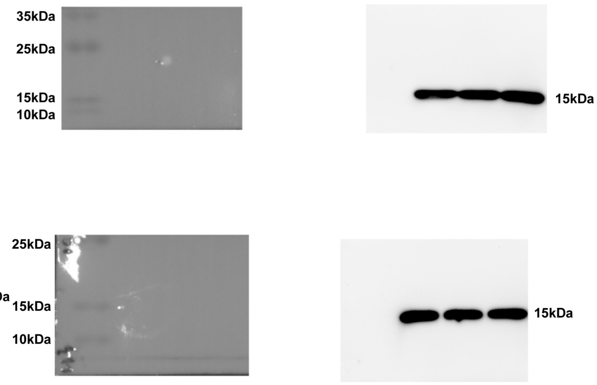

SREBF1-Cytoplasm

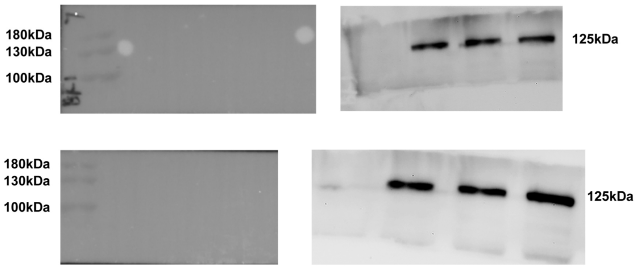

SREBF 2-Cytoplasm

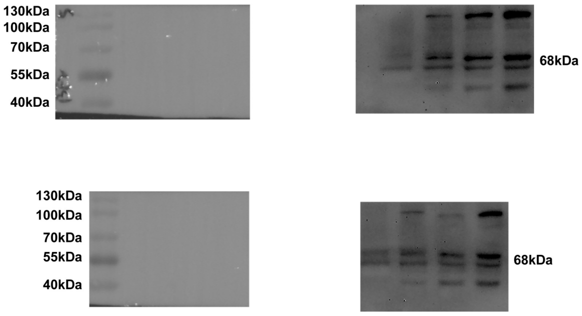

β-actin-Cytoplasm

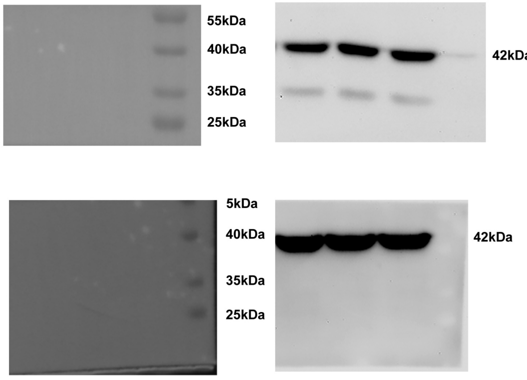

SREBF1-Lysate

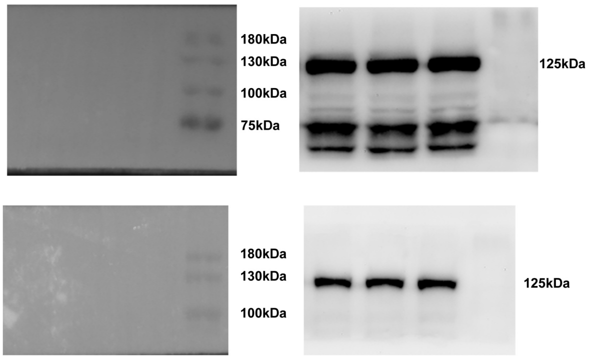

SREBF 2-Lysate

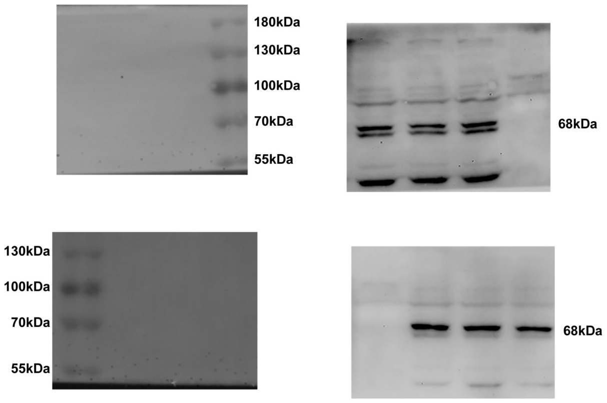

β-actin-Lysate

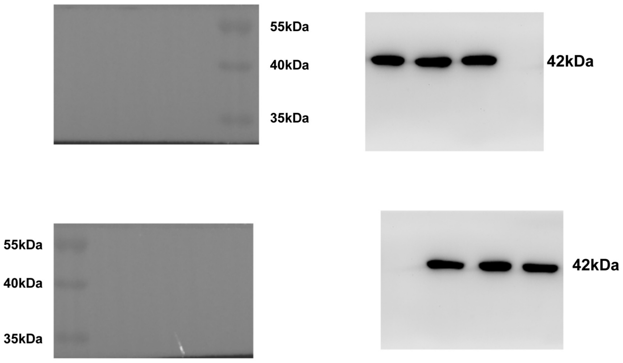

Figure 6C

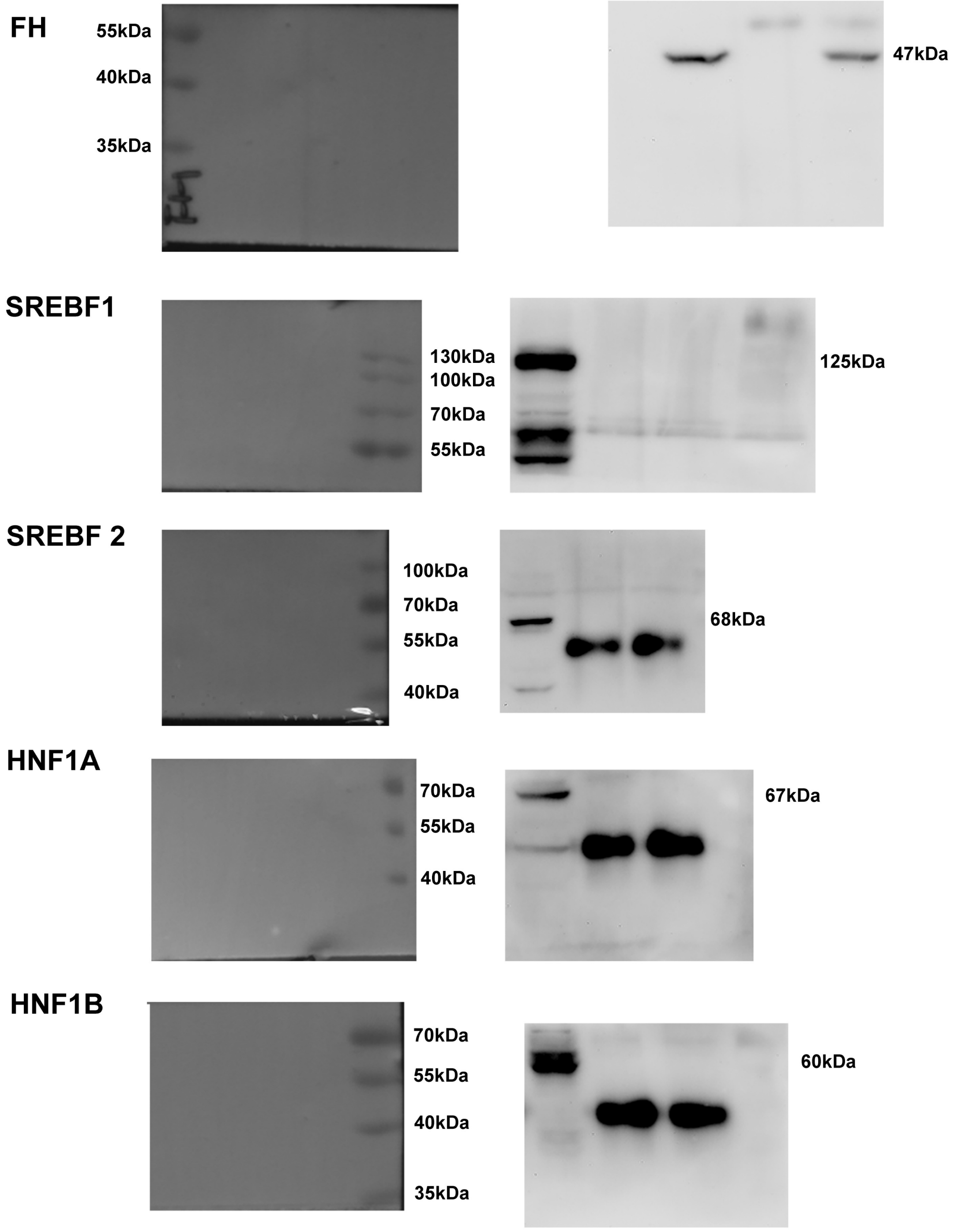
FH
55kDa
40kDa
35kDa
47kDa
SREBF1
130kDa
100kDa
70kDa
55kDa
125kDa
SREBF 2
100kDa
70kDa
55kDa
40kDa
68kDa
HNF1A
70kDa
55kDa
40kDa
67kDa
HNF1B
70kDa
55kDa
40kDa
35kDa
60kDa

Figure 6D

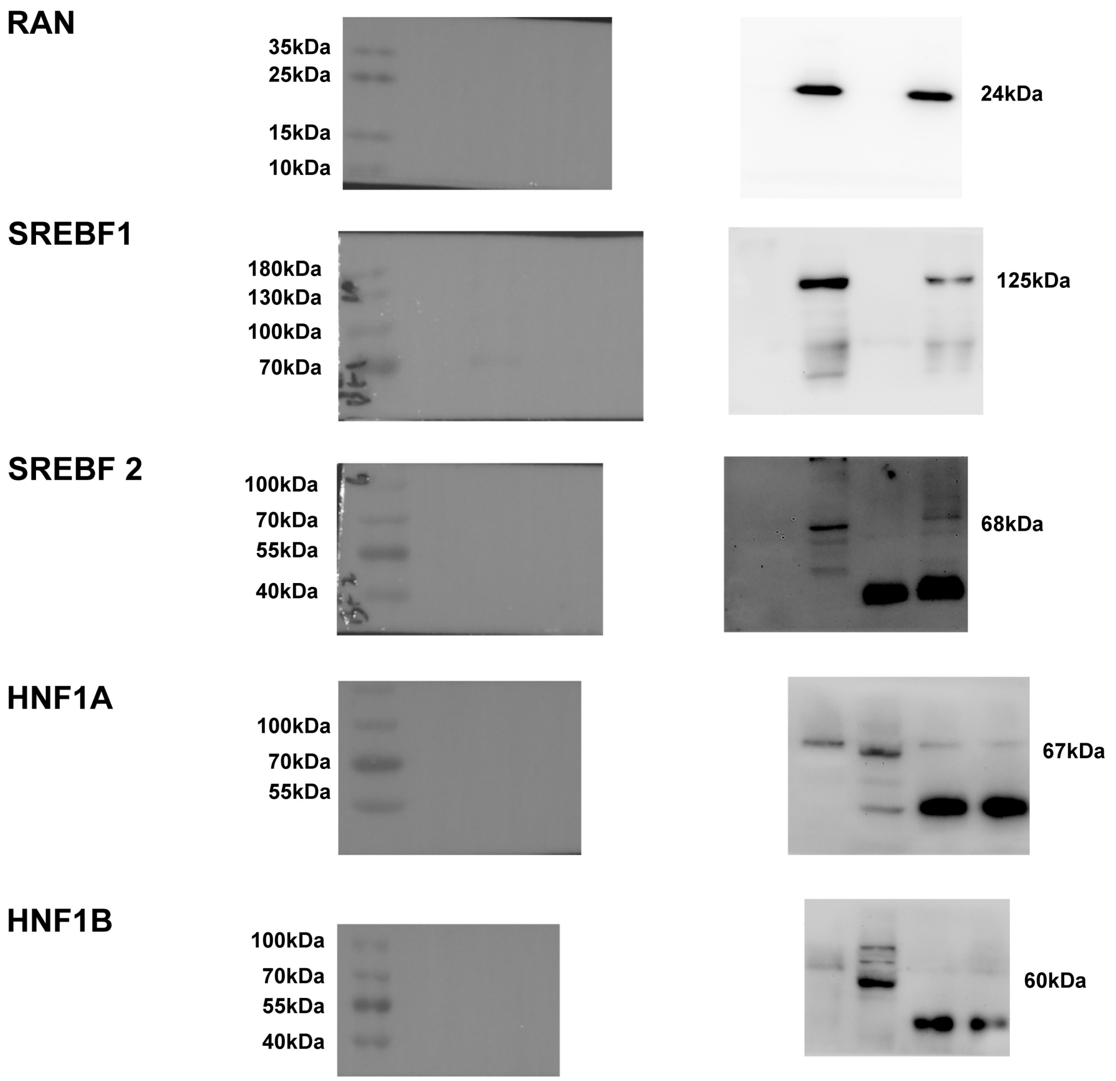
RAN
35kDa
25kDa
15kDa
10kDa
24kDa
SREBF1
180kDa
130kDa
100kDa
70kDa
125kDa
SREBF 2
100kDa
70kDa
55kDa
40kDa
68kDa
HNF1A
100kDa
70kDa
55kDa
67kDa
HNF1B
100kDa
70kDa
55kDa
40kDa
60kDa

Figure 6E

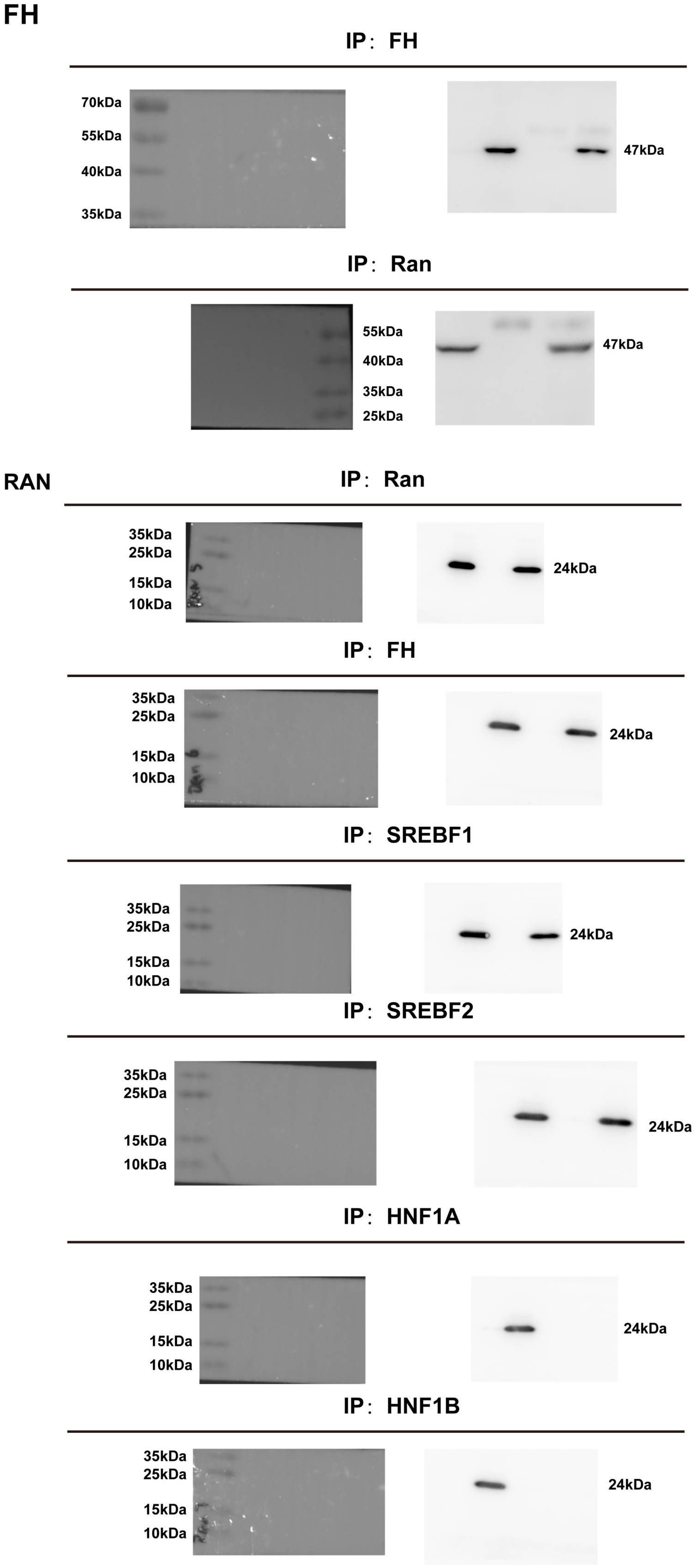
FH
IP：FH
70kDa
55kDa
40kDa
35kDa
47kDa
IP：Ran
55kDa
40kDa
35kDa
25kDa
47kDa
RAN
IP：Ran
35kDa
25kDa
15kDa
10kDa
24kDa
IP：FH
35kDa
25kDa
15kDa
10kDa
24kDa
IP：SREBF1
35kDa
25kDa
15kDa
10kDa
24kDa
IP：SREBF2
35kDa
25kDa
15kDa
10kDa
24kDa
IP：HNF1A
35kDa
25kDa
15kDa
10kDa
24kDa
IP：HNF1B
35kDa
25kDa
15kDa
10kDa
24kDa

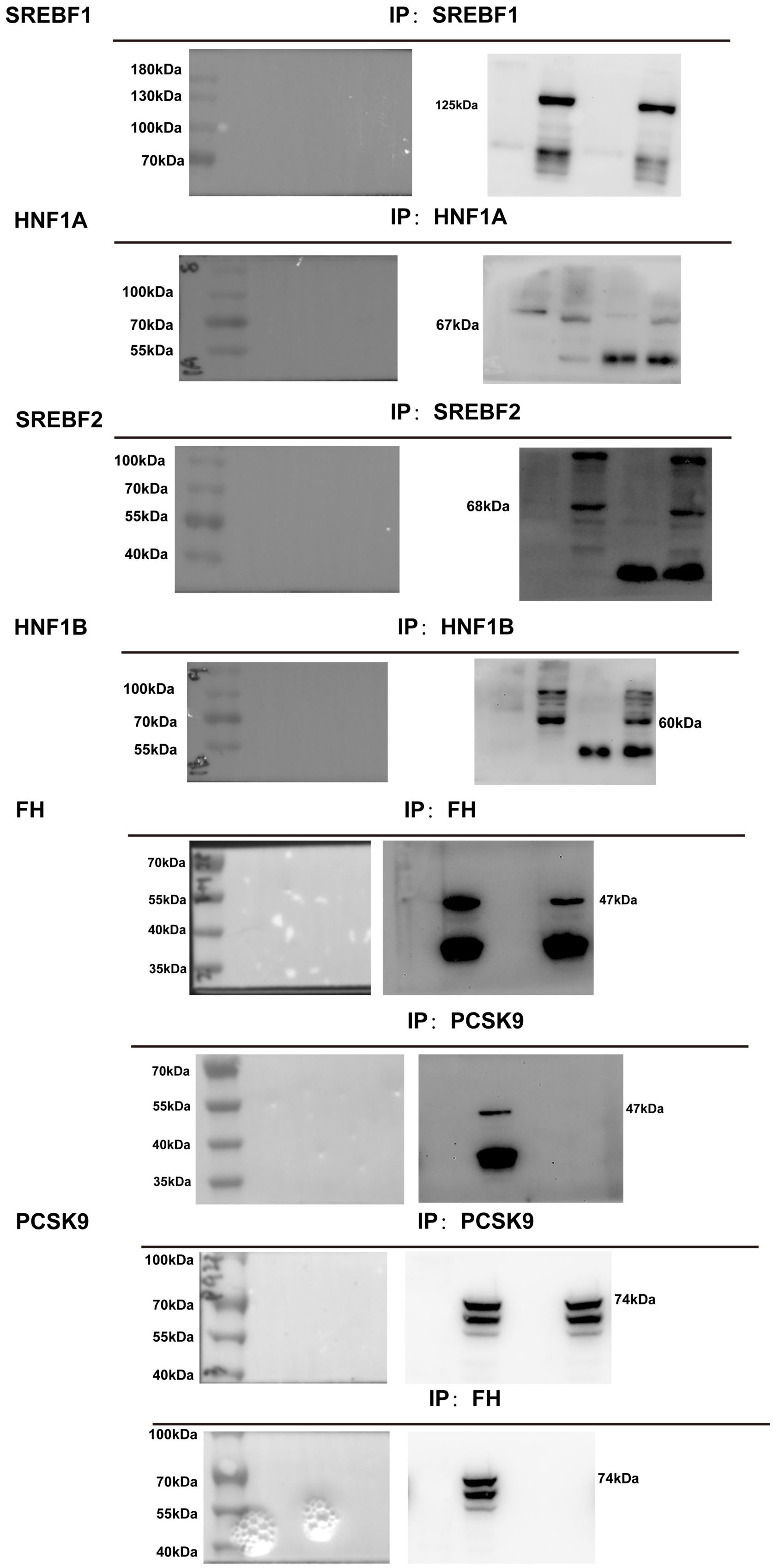
SREBF1
IP: SREBF1
180kDa
130kDa
100kDa
70kDa
125kDa
HNF1A
IP: HNF1A
100kDa
70kDa
55kDa
67kDa
SREBF2
IP: SREBF2
100kDa
70kDa
55kDa
40kDa
68kDa
HNF1B
IP: HNF1B
100kDa
70kDa
55kDa
60kDa
FH
IP: FH
70kDa
55kDa
40kDa
35kDa
47kDa
IP: PCSK9
70kDa
55kDa
40kDa
35kDa
47kDa
PCSK9
IP: PCSK9
100kDa
70kDa
55kDa
40kDa
74kDa
IP: FH
100kDa
70kDa
55kDa
40kDa
74kDa

Figure 6F

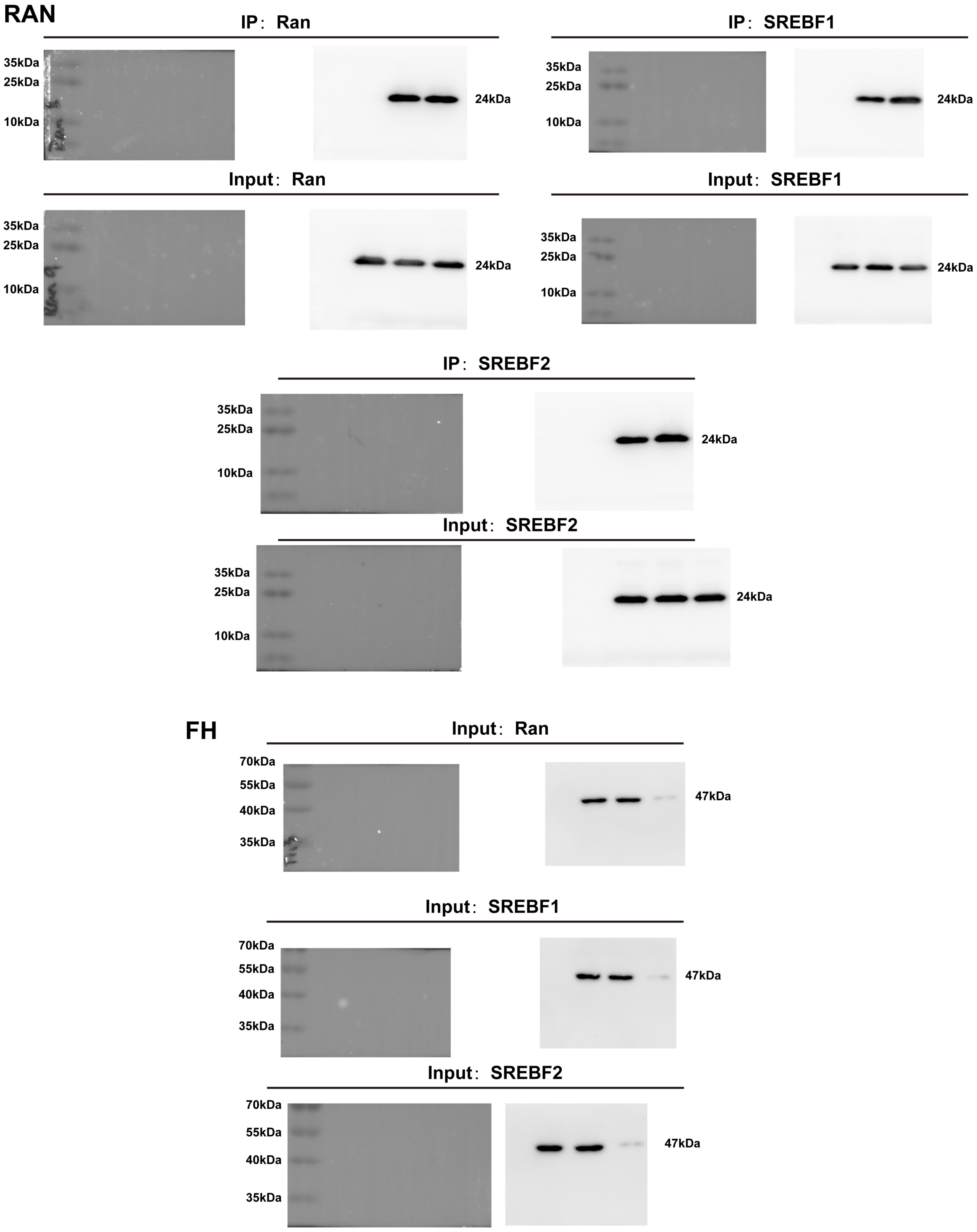
RAN
IP: Ran
35kDa
25kDa
10kDa
24kDa
IP: SREBF1
35kDa
25kDa
10kDa
24kDa
Input: Ran
35kDa
25kDa
10kDa
24kDa
Input: SREBF1
35kDa
25kDa
10kDa
24kDa
IP: SREBF2
35kDa
25kDa
10kDa
24kDa
Input: SREBF2
35kDa
25kDa
10kDa
24kDa
FH
Input: Ran
70kDa
55kDa
40kDa
35kDa
47kDa
Input: SREBF1
70kDa
55kDa
40kDa
35kDa
47kDa
Input: SREBF2
70kDa
55kDa
40kDa
35kDa
47kDa

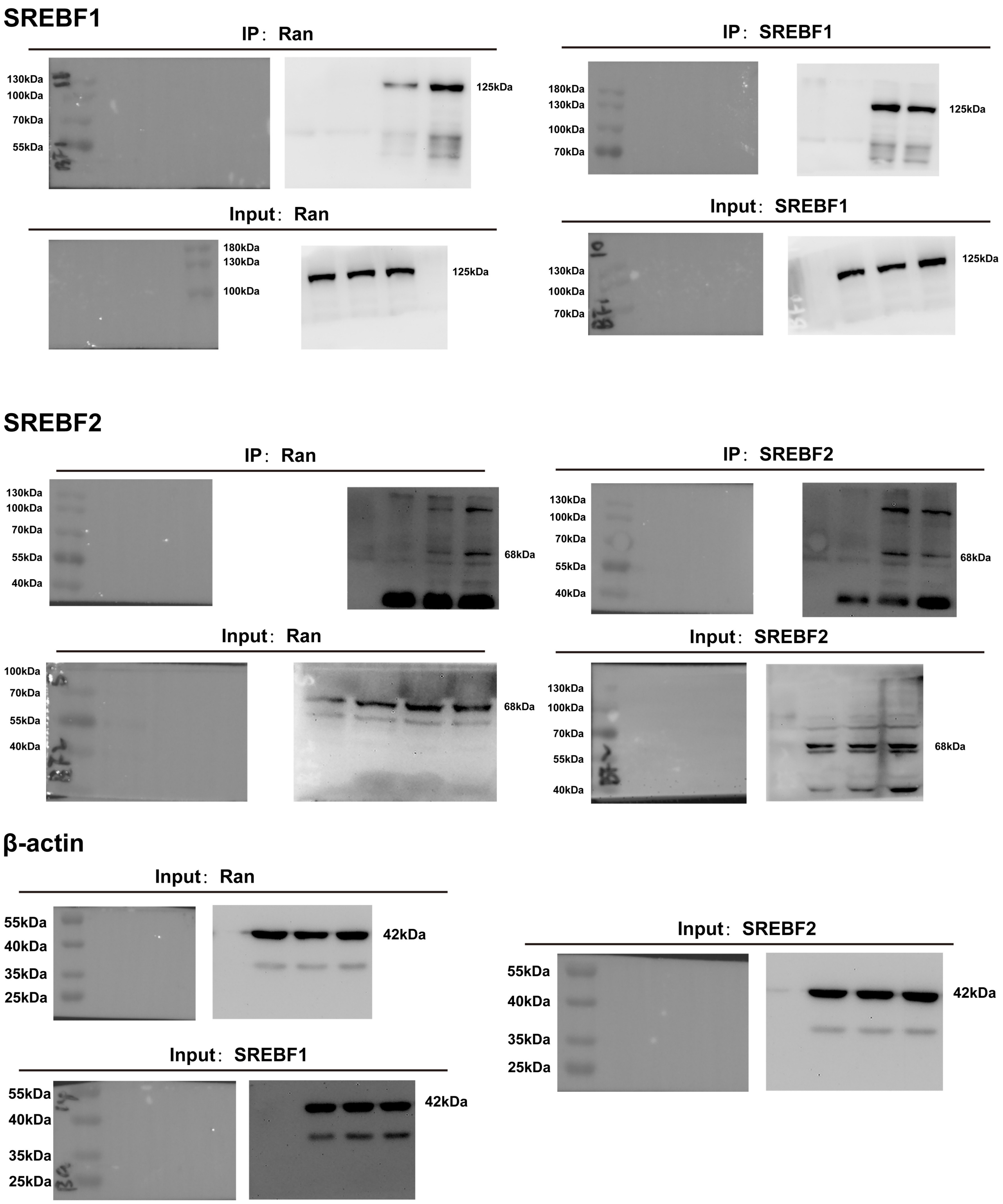
SREBF1
IP: Ran
130kDa
100kDa
70kDa
55kDa
125kDa
IP: SREBF1
180kDa
130kDa
100kDa
70kDa
125kDa
Input: Ran
180kDa
130kDa
100kDa
125kDa
Input: SREBF1
130kDa
100kDa
70kDa
125kDa
SREBF2
IP: Ran
130kDa
100kDa
70kDa
55kDa
40kDa
68kDa
IP: SREBF2
130kDa
100kDa
70kDa
55kDa
40kDa
68kDa
Input: Ran
100kDa
70kDa
55kDa
40kDa
68kDa
Input: SREBF2
130kDa
100kDa
70kDa
55kDa
40kDa
68kDa
β-actin
Input: Ran
55kDa
40kDa
35kDa
25kDa
42kDa
Input: SREBF2
55kDa
40kDa
35kDa
25kDa
42kDa
Input: SREBF1
55kDa
40kDa
35kDa
25kDa
42kDa

Figure 6G

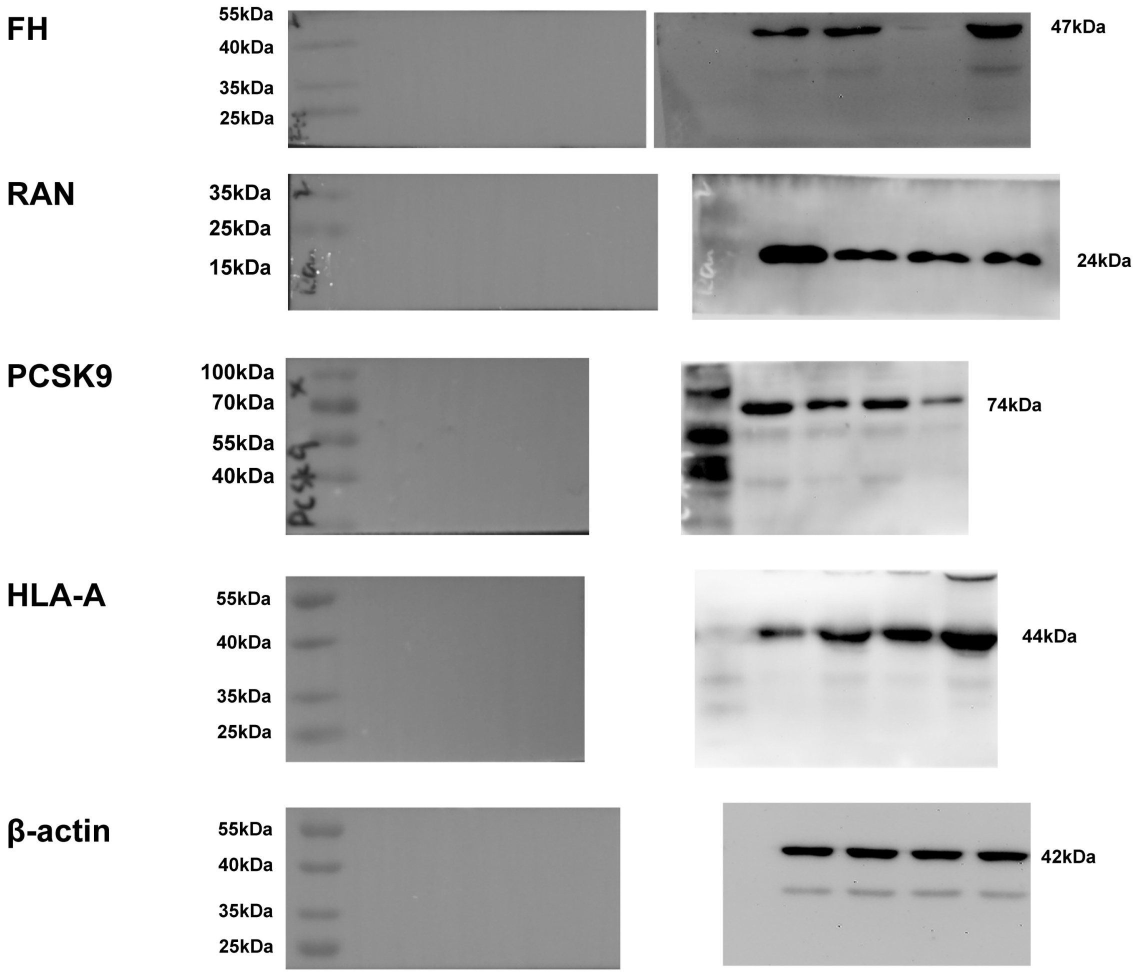
FH
55kDa
40kDa
35kDa
25kDa
47kDa
RAN
35kDa
25kDa
15kDa
24kDa
PCSK9
100kDa
70kDa
55kDa
40kDa
74kDa
HLA-A
55kDa
40kDa
35kDa
25kDa
44kDa
β-actin
55kDa
40kDa
35kDa
25kDa
42kDa

Figure S2A

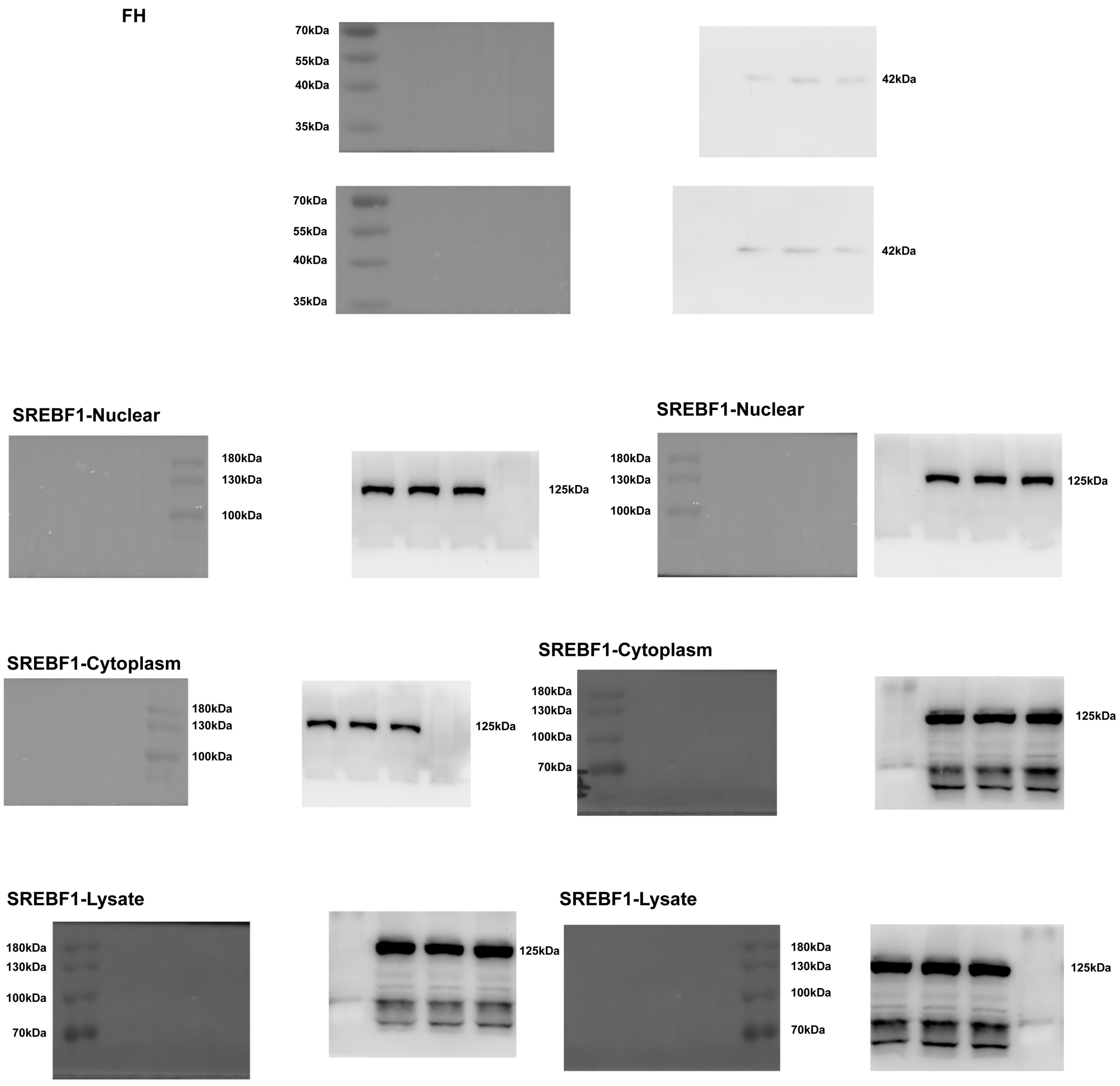
FH
70kDa
55kDa
40kDa
35kDa
42kDa
70kDa
55kDa
40kDa
35kDa
42kDa
SREBF1-Nuclear
180kDa
130kDa
100kDa
125kDa
SREBF1-Nuclear
180kDa
130kDa
100kDa
125kDa
SREBF1-Cytoplasm
180kDa
130kDa
100kDa
125kDa
SREBF1-Cytoplasm
180kDa
130kDa
100kDa
70kDa
125kDa
SREBF1-Lysate
180kDa
130kDa
100kDa
70kDa
125kDa
SREBF1-Lysate
180kDa
130kDa
100kDa
70kDa
125kDa

SREBF2-Nuclear

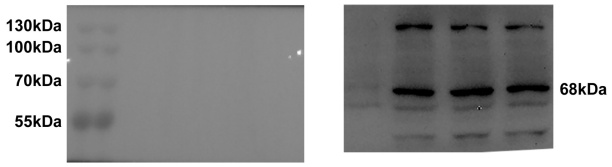

SREBF2-Nuclear

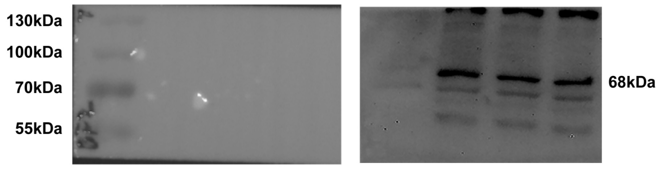

SREBF2-Cytoplasm

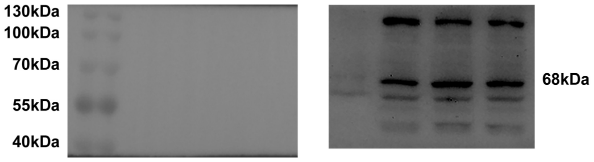

SREBF2-Cytoplasm

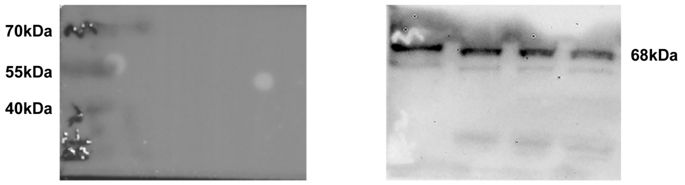

SREBF2-Lysate

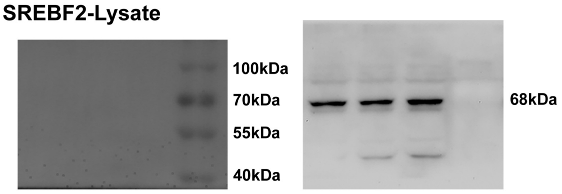

SREBF2-Lysate

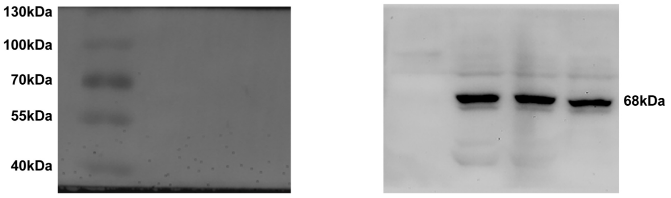

H3-Nuclear

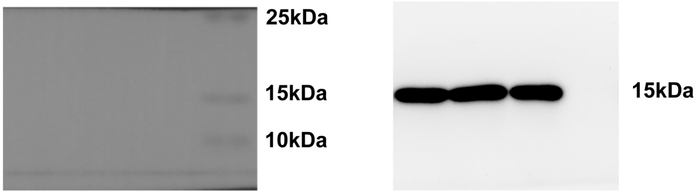

H3-Nuclear

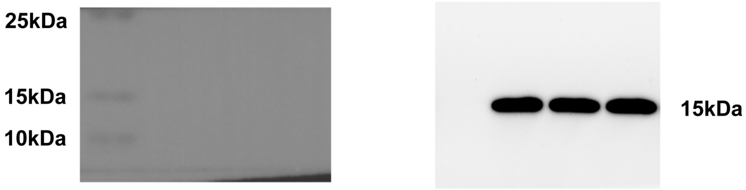

β-actin-Cytoplasm

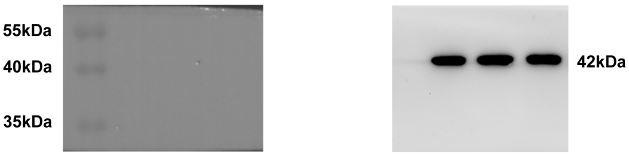

β-actin-Cytoplasm

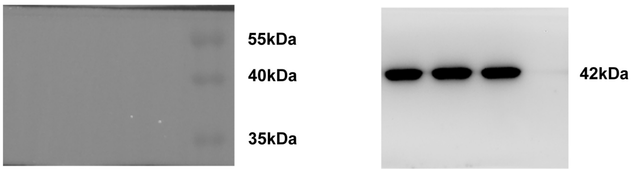

β-actin-Lysate

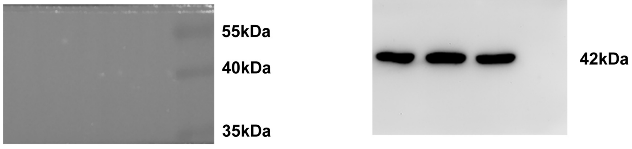

β-actin-Lysate

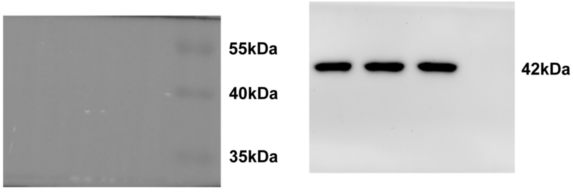

Figure S3A

FH

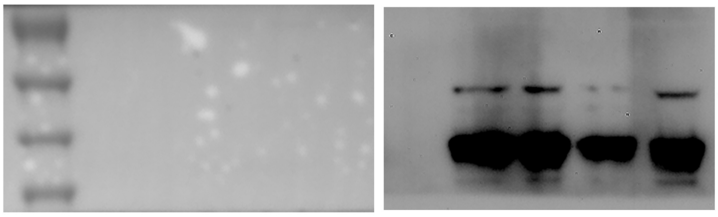

β-actin

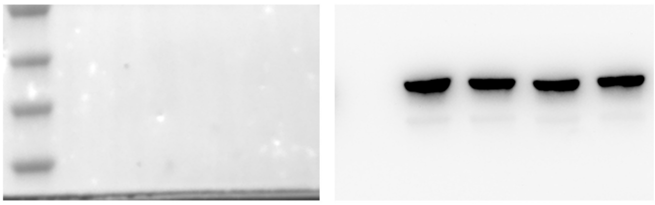

Supplement: Supplementary file 1 [file cancers-16-00713-s001.zip › cancers-2799512-supplementary/supplementary/Original image.pdf]
