# Supplementary material for: Fumarate Hydratase Enhances the Therapeutic Effect of PD-1 Antibody in Colorectal Cancer by Regulating PCSK9
Source: Cancers (Basel). 2024 Feb 8;16(4):713. doi: 10.3390/cancers16040713 (PMC10887080; doi:10.3390/cancers16040713)
Supplement: Supplementary file 1 [file cancers-16-00713-s001.zip › cancers-2799512-supplementary/supplementary/Supplementary Materials.pdf]

FH enhances the immunotherapy effect of colorectal cancer by inhibiting ran-mediated nuclear import of PCSK9 transcription factor

Le Qin, Liang Shi, Yu Wang, Haxin Yu, Zhouyuan Du, Mian Chen, Yuxuan Cai, Yinghao Cao, Shenghe Deng, Jun Wang, Denglong Cheng, Yixin Heng, Jiaxin Xu, Ke Wu, Kailin Cai

Figure S1. Fumaric acid does not significantly regulate PCSK9 expression.

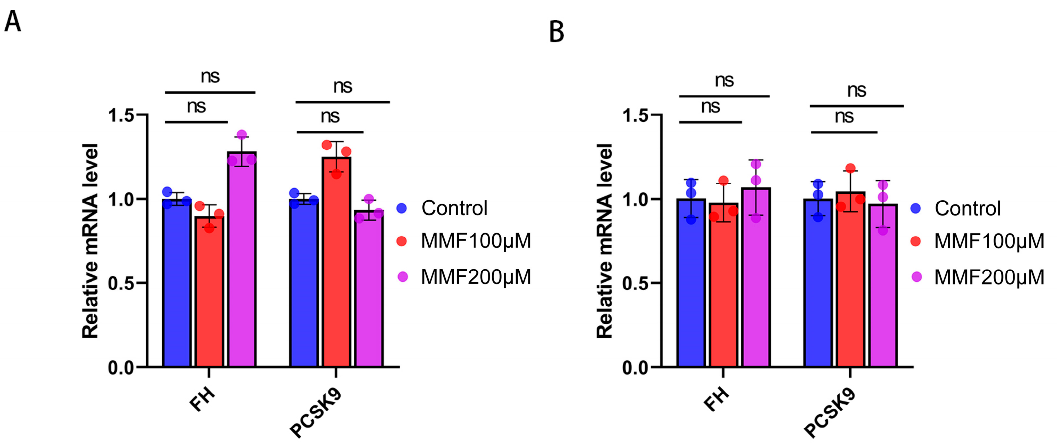

(A) SW620 cells, treated with 100 μM or 200 μM Monomethyl fumarate (MMF), followed by assessment of FH and PCSK9 mRNA levels through RT-qPCR after 24 hours. (B) SW620 cells with knocked-down FH, treated with 100 μM or 200 μM MMF, followed by assessment of FH and PCSK9 mRNA levels through RT-qPCR after 24 hours. Relative ns, no significance.

Figure S2. FA does not have a significant effect on the nuclear translocation of SREBF1/2.

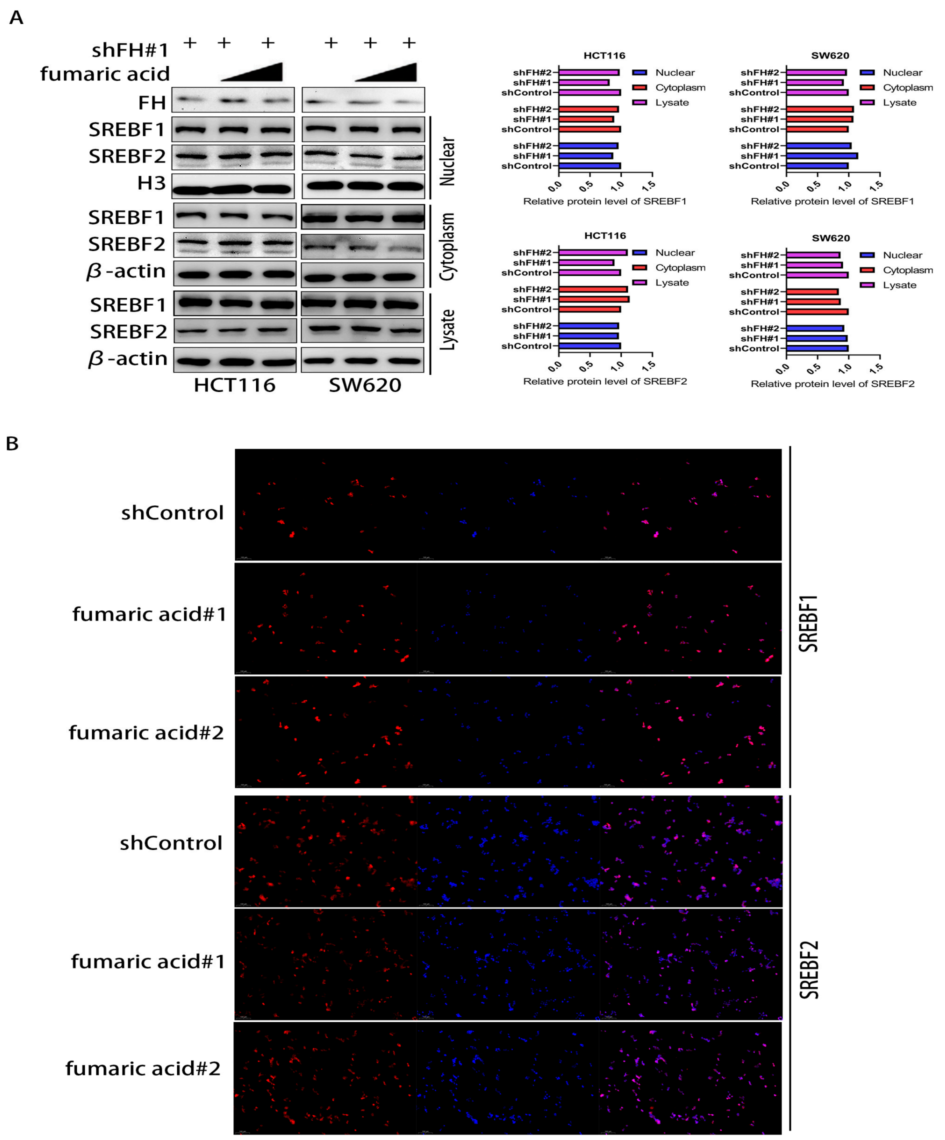

(A) Stable cell lines with knocked-down FH were established in HCT116 and SW620 cells. Subsequently, 100 μM or 200 μM MMF was added to the FH-knocked-down cell lines, and after 72 hours, the effect of FA addition on the expression of SREBF1 and SREBF2 in the nucleus and cytoplasm was analyzed using Western blot. (B) In HCT116 cells, after knocking down FH, the localization of

SREBF1 and SREBF2 proteins was determined using immunofluorescence.

Figure S3. The proliferative capacity of the cells after re-overexpression of FH when FH was knockdown.

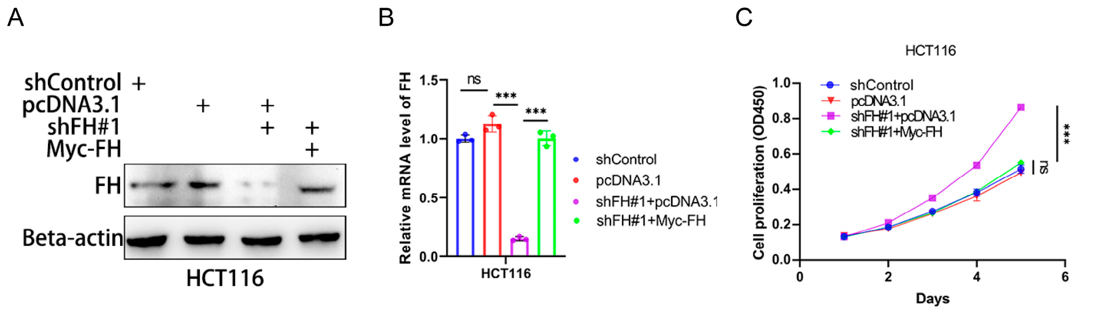

(A) The protein levels of FH ain HCT116 and SW620 cells with FH knockdown or overexpression were detected by Western blotting. (B) RT-qPCR was used to determine the transfection efficiency of gene knockdown or overexpression. (C) Growth curves of HCT116 cells after FH knockdown or overexpression were plotted using CCK-8 analysis ($n = 3$). Relative *** $p < 0.001$.

Supplementary Materials and Methods

Public datasets for data mining and bioinformatics analysis

1) TCGA-COAD: Transcriptomic data and clinical information for COAD patients were sourced from the GDC data portal (https://portal.gdc.cancer.gov/). Data for 275 COAD patients and 60,488 genes were retrieved. Among these patients, 41 samples had

matched normal tissues.

2) TCGA-READ: Transcriptomic data and clinical information for READ patients were obtained from the GDC data portal (https://portal.gdc.cancer.gov/). Data for 92 READ patients and 60,488 genes were collected. Within these patients, 10 samples had matched normal tissues.

3) TCGA TARGET GTEx: Aggregated transcriptomic data and clinical information for TCGA CRC samples, and GTEx normal colorectal samples came from the TCGA TARGET GTEx dataset on the Xena platform (http://xena.ucsc.edu/). Data for 380 CRC patients and 60,499 genes were obtained. Among these patients, 51 samples had matched normal tissues. Additionally, this dataset also includes colorectal samples from 308 healthy individuals.

Survival analysis

CRC patients were divided into two groups based on the median expression levels of key genes. The differences in overall survival (OS) between the high-expression group and the low-expression group were assessed using the Kaplan-Meier method, followed by a log-rank test.

GSEA of differentially expressed genes

The input genes for GSEA are sorted based on the logFC values of differentially expressed genes. The signaling pathways activated or inhibited by key genes are determined by the NES values derived from GSEA.

Identification of transcription factors

The transcription factors of key proteins were obtained through enrichment analysis in the ChIP-Atlas database (https://chip-atlas.org/). Subsequently, the ChIP-seq tracks of the transcription factors were visualized using IGV, and validation was conducted with ChIP-qPCR.

PPI network of key proteins

The PPI network of key proteins was constructed using STRING v11.0 (https://string-db.org/).

Statistical analysis and visualization

Microsoft R Open v4.0.2 was used for transcriptomic data mining, bioinformatics analysis, and visualization. IGV v2.16.2 was utilized for the analysis and visualization of ChIP-seq data. STRING v12.0 was employed for the analysis and visualization of the PPI network.

Table S1. The list of shRNA sequence.

shRNA	Sequence
shFH-RNA control	TTCTCCGAACGTGTCACGT
shFH-RNA 1#	ccCAACGATCATGTTAATAAA
shFH-RNA 2#	cgCTGAAGTAAACCAGGATTA
shFh1-RNA control	TTCTCCGAACGTGTCACGT
shFh1-RNA	ATTGAAGGTTCCAACCGATAA
shPCSK9-RNA control	TTCTCCGAACGTGTCACGT
shPCSK9-RNA	GCCGTAGACAACACGTGTGTA
shRAN-RNA control	TTCTCCGAACGTGTCACGT
shRAN-RNA	GCACAGTATGAGCACGACTTA

Table S2. The list of primer sequence.

Gene	Sequence	Application	length
Beta-actin (Human)	Forward: CCTTCCTGGGCATGGAGTC	RT-qPCR	19
	Reverse: TGATCTTCATTGTGCTGGGTG		21
FH (Human)	Forward: GGAGGTGTGACAGAACGCAT	RT-qPCR	20
	Reverse:		22

	CATCTGCTGCCTTCATTATTGC		
Beta-actin (Mouse)	Forward: TGCTGTCCCTGTATGCCTCTG	RT-qPCR	21
	Reverse: TGATGTCACGCACGATTTCC		20
Fh1 (Mouse)	Forward: GAATGGCAAGCCAAAATTCCTT	RT-qPCR	22
	Reverse: TCTTACGGTCTGAGCACCATAA		22
PCSK9 (Human)	Forward: AGACCCACCTCTCGCAGTC	RT-qPCR	19
	Reverse: GGAGTCCTCCTCGATGTAGTC		21
RAN (Human)	Forward: TCTGGCTTGCTAGGAAGCTCA	RT-qPCR	21
	Reverse: GCTGGGTCCATGACAACTTCT		21
Primer Outside	Forward: GAATGGTGGGTGTACATCGCT	ChIP-qPCR	21
	Reverse: AATCATTGTGCAAACGGAGAGC		22
Primer Inside	Forward:	ChIP-qPCR	20

	GAGGCCGAAACCTGATCCTC<br>Reverse:<br>GTCGCTGCGGAAACCTTCTA	20

Table S3. The list of antibodies.

Antibody	Catalogue	Dilution	Application
Beta-Actin	66009-1-Ig, Proteintech	1:20000	WB
FH	10966-1-AP, Proteintech	1:1000	WB
MYC	60003-2-Ig, Proteintech	1:2000	WB
PCSK9	27882-1-AP, Proteintech	1:1000	WB
SREBF1	14088-1-AP, Proteintech	1:2000	WB
SREBF2	A01678-2, BOSTER	1:1000	WB
Histone H3	68345-1-Ig, Proteintech	1:5000	WB
RAN	10469-1-AP, Proteintech	1:1000	WB
HLA-A	15240-1-AP, Proteintech	1:500	WB
Beta-Actin	66009-1-Ig, Proteintech	1:20000	IP
FH	10966-1-AP, Proteintech	1:1000	IP
SREBF1	14088-1-AP, Proteintech	1:2000	IP
SREBF2	A01678-2, BOSTER	1:1000	IP
HNF1A	22426-1-AP, Proteintech	1:1000	IP
HNF1B	12533-1-AP, Proteintech	1:1000	IP

RAN	10469-1-AP, Proteintech	1:1000	IP
PCSK9	55206-1-AP, Proteintech	1:1000	IP
IgG (rabbit)	A7016, Beyotime	1:1000	IP
IgG (mouse)	A7028, Beyotime	1:2000	IP
FH	10966-1-AP, Proteintech	1:100	IHC
PCSK9	55206-1-AP, Proteintech	1:500	IHC
CD8 alpha	ab217344, abcam	1:400	IHC
FOXP3	ab215206, abcam	1:100	IHC
PD-L1	28076-1-AP, Proteintech	1:500	IHC
SREBF1	14088-1-AP, Proteintech	1:50	ChIP
SREBF2	ab112046, Abcam	1:100	ChIP
HNF1A	22426-1-AP, Proteintech	1:1000	ChIP
HNF1B	12533-1-AP, Proteintech	1:1000	ChIP
SREBF1	14088-1-AP, Proteintech	1:50	IF
SREBF2	A01678-2, BOSTER	1:50	IF
